# A proteomic analysis of C-reactive protein stimulated THP-1 monocytes

**DOI:** 10.1186/1477-5956-9-1

**Published:** 2011-01-10

**Authors:** Steffen U Eisenhardt, Jonathon Habersberger, Karen Oliva, Graeme I Lancaster, Mustafa Ayhan, Kevin J Woollard, Holger Bannasch, Greg E Rice, Karlheinz Peter

**Affiliations:** 1 Baker IDI Heart and Diabetes Institute, Melbourne, Victoria, Australia; 2 Department of Plastic and Hand Surgery, University of Freiburg Medical Centre, Freiburg, Germany; 3 Department of Obstetrics and Gynaecology, University of Melbourne, Victoria, Australia

## Abstract

**Background:**

C-reactive protein (CRP) is a predictor of cardiovascular risk. It circulates as a pentameric protein in plasma. Recently, a potential dissociation mechanism from the disc-shaped pentameric CRP (pCRP) into single monomers (monomeric or mCRP) has been described. It has been shown that mCRP has strong pro-inflammatory effects on monocytes. To further define the role of mCRP in determining monocyte phenotype, the effects of CRP isoforms on THP-1 protein expression profiles were determined. The hypothesis to be tested was that mCRP induces specific changes in the protein expression profile of THP-1 cells that differ from that of pCRP.

**Methods:**

Protein cell lysates from control and mCRP, pCRP or LPS-treated THP-1 cells were displayed using 2-dimensional SDS PAGE and compared. Differentially expressed proteins were identified by MALDI-TOF MS and confirmed by Western blotting.

**Results:**

mCRP significantly up-regulates ubiquitin-activating enzyme E1, a member of the ubiquitin-proteasome system in THP-1 monocytes. Furthermore, HSP 70, alpha-actinin-4 (ACTN4) and alpha-enolase/enolase 1 were upregulated. The proteomic profile of LPS and pCRP treated monocytes differ significantly from that of mCRP.

**Conclusion:**

The data obtained in this study support the hypothesis that isoform-specific effects of CRP may differentially regulate the phenotype of monocytes.

## Background

CRP is an acute phase reactant and member of the evolutionary highly conserved pentraxin family. As such, it consists of five non-covalently linked subunits of ~23 kDa. CRP can exist in at least two different conformations, as a cyclic, disc-shaped pentamer of 115 kDa (pCRP) that is circulating in plasma and as a monomeric conformation termed modified or monomeric CRP (mCRP). As a major source of mCRP, *in vivo*, we recently identified a mechanism of pCRP dissociation localized on activated platelets and cells exposing bioactive lipids, such as lysophosphatidylcholine [1].

pCRP and mCRP exhibit very distinct biological activities. Both CRP forms, however, display pro-inflammatory effects. mCRP was found to be a considerably more potent activator of endothelial cells and monocytes than pCRP [1,2]. pCRP and mCRP exert opposing effects on neutrophil trafficking into tissues [3] and platelet and thrombus growth [4,5]. Other studies found that pCRP induces pro-inflammatory cytokine release from endothelial cells and monocytes and evokes endothelial dysfunction and monocyte adhesion to the endothelium [6].

Recently, we demonstrated that mCRP, but not pCRP, is enriched in atherosclerotic plaques and is derived from a localized dissociation process of the circulating pCRP mediated by activated and apoptotic cell-membranes [1]. This leads to a localized activation of inflammatory cells, such as monocytes and the production of reactive oxygen species within monocytes. Monocytes are central in the pathogenesis of atherosclerosis. Lipids are deposited on and in arterial walls causing inflammation of the intima, followed by monocyte infiltration, leading to wall thickening and formation of atherosclerotic plaques [7]. The interaction of monocytes with proteins enriched in the plaque is of particular interest as improvements in our understanding of this process may enable the identification of novel therapeutic targets. These could potentially inhibit the uptake of lipoproteins and the release of reactive oxygen species and immune mediators by monocyte-derived macrophages. This process leads to the formation of foam cells that collectively contribute to atheroma formation. The proteomic changes of THP-1 monocytes induced by CRP isoforms have not been identified to date. In this study, the hypothesis to be tested was that mCRP induces specific changes in the protein expression profile of THP-1 cells that promotes a pro-inflammatory phenotype. This hypothesis was tested in an *in vitro *treatment - control experimental design in which the effects of CRP isoforms and LPS on protein expression profiles were determined.

## Results

**Figure 1:**
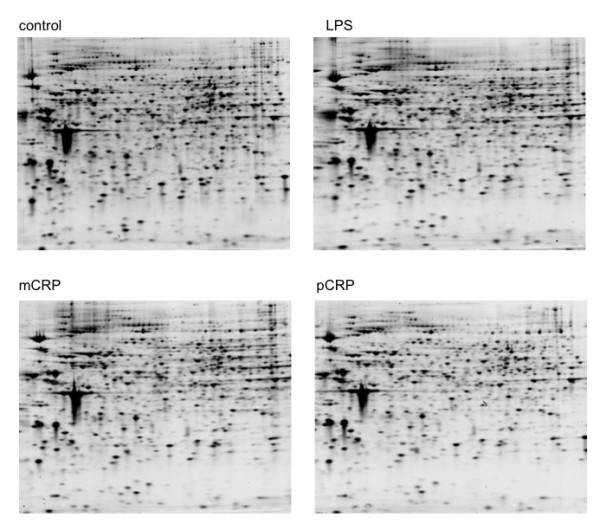
**Protein expression of PBS, LPS, mCRP and pCRP-treated THP-1 monocytes**. Proteins from whole cell lysates were separated on a pH 3-10 IPG strip in the first dimension and on a SDS-polyacrylamide (12%) gel in the second dimension. Representative Coomassie-stained gels for each treatment group are shown.

**Figure 2:**
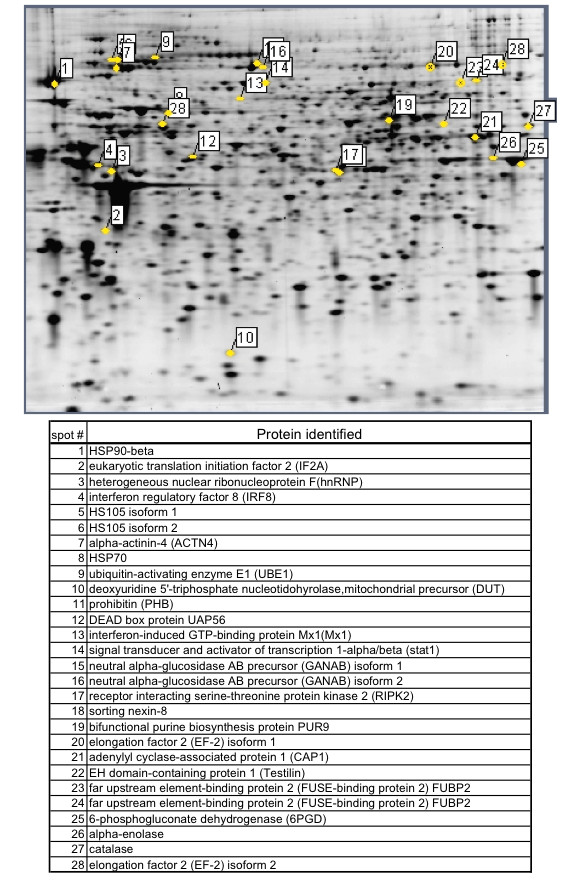
**Protein expression map of THP-1 monocytes**. Spots that are differentially expressed in the three treatment groups were analysed by MALDI-ToF. Protein identity of these proteins is given in the figure.

**Figure 3:**
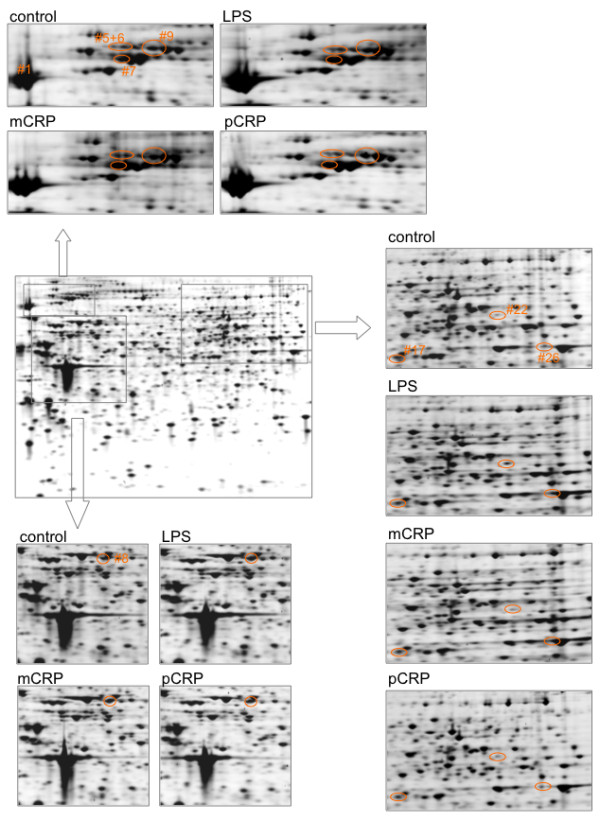
**High-magnification areas of proteins differentially expressed**. The middle section shows a representative Coomassie-stained gel of proteins derived from control cells. Around the typical control gel, enlarged areas of interest derived from control cells, LPS, mCRP and pCRP treated cells are shown. Protein spots of interests are encircled. The spot numbers are identical to those given in Figure 2.

**Figure 4:**
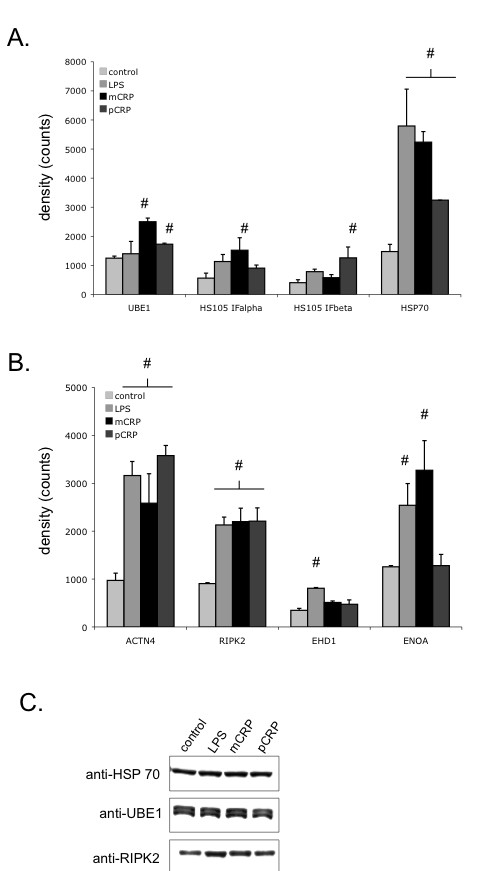
**Quantitative analysis of differentially expressed proteins**. **A. mCRP induces expression of cellular stress related proteins of the ubiquitin/proteasome system and the heat shock response.** Expression levels of several proteins of interest were quantified and are given in the graphs as mean +/- standard deviation. # indicates p < 0.05 compared to control. mCRP up-regulates UBE1, and HSP 70, as well as the alpha isoform of HSP 105. pCRP induces the beta isoform of HSP105. **B. Differential expression of proteins involved in the inflammatory response. **ACTN4 and RIPK2 are increased in the LPS, as well as in the m- and pCRP group. ENOA is increased by LPS and mCRP stimulation of THP-1 monocytes, whereas EHD1 is significantly upregulated after stimulation with LPS. **C. Western blot analysis of selected proteins that are differentially expressed in THP-1 cells. **Selected Proteins were validated by Western blotting. Western blot results are shown exhibiting specific staining of 1D-gels with anti-HSP70, anti-RIPK2 and anti-UBE1 antibodies. Analysis served as a confirmation of the protein identity in addition to the Mass-spec analysis.

A 2D-SDS PAGE display of cell lysates from THP-1 cells is presented in Figure 1. Overall 12 gels were analyzed, for each treatment in triplicates, 493 to 508 protein spots were displayed. When displays from mCRP and pCRP-treated THP-1 monocytes were compared 15 spots had at least 2-fold difference between pCRP and mCRP and were further identified (Figure 1). Table 1 presents differentially expressed proteins that change > 2-fold in expression levels. Spots that were identified by MALDI ToF mass spectrometry are presented in Figure 2. Figure 3 presents high magnification views of areas of interest that changed significantly compared to the control group. Most notably proteins of the ubiquitin-proteasome system and of the heat shock response are up-regulated by mCRP. Specific proteins of interest are listed in Table 2 with mean ± standard deviation, which is also graphically shown in Figure 4. mCRP increases the expression of UBE1, HSP 105, IF alpha and HSP 70 significantly when compared to control THP-1 cells, suggesting increased cellular stress in this treatment group. pCRP induces HSP 105 isoform beta expression, as well as HSP 70 expression, albeit weaker than mCRP. Interestingly, only LPS induces a significantly increased expression of HSP 90.

**Table 1:** Differentially expressed proteins in LPS-, mCRP- or pCRP-treated THP-1 monocytes.

#	Protein identified	MW	pI	MOWSE score	**Cov**.	LPS	mCRP	pCRP
1	HSP90-beta	83554	4,97	162	40%	1,46	0,38	1,06

2	eukaryotic translation initiation factor 2(IF2A)	36374	5,02	76	27%	0,48	1,46	0,33

3	heterogeneous nuclear ribonucleoprotein F(hnRNP)	45985	5,38	65	18%	0,77	2,3	2,88

4	interferon regulatory factor 8 (IRF8)	49123	6,38	64	26%	0,35	0,32	0,48

5	HS105 isoform 1	97716	5,28	58	11%	2,02	2,71	1,61

6	HS105 isoform 2	97716	5,28	90	19%	1,94	1,43	3,1

7	alpha-actinin-4 (ACTN4)	105245	5,27	55	16%	3,26	2,66	3,69

8	HSP70	70280	5,48	75	18%	3,93	3,55	2,2

9	ubiquitin-activating enzyme E1 (UBE1)	118858	5,49	99	22%	1,12	2	1,39

10	deoxyuridine 5'-triphosphate nucleotidohyrolase (DUT)	26975	9,65	70	28%	0,69	0,85	0,46

11	prohibitin (PHB)	29843	5,57	94	44%	36,16	35,12	30,07

12	DEAD box protein UAP56	49416	5,44	72	22%	0,73	0,6	2,32

13	interferon-induced GTP-binding protein Mx1 (Mx1)	75886	5,6	85	22%	2,09	1,14	1,19

14	signal transducer and activator of transcription 1- alpha/beta (stat1)	87850	5,74	104	24%	2,06	1,2	1,24

15	neutral alpha-glucosidase AB precursor (GANAB) isoform 1	107263	5,74	114	20%	0,77	0,44	0,9

16	neutral alpha-glucosidase AB precursor (GANAB) isoform 2	107263	5,74	58	13%	1,75	4,56	1,3

17	receptor interacting serine-threonine protein kinase 2 (RIPK2)	61726	6,63	59	11%	2,35	2,43	2,44

18	sorting nexin-8	52935	6,96	55	16%	0,26	0,25	0,28

19	bifunctional purine biosynthesis protein (PUR9)	65089	6,27	85	29%	2,17	1,55	1,27

20	elongation factor 2 (EF-2) isoform 1	96246	6,41	129	23%	0,6	0,97	0,34

21	adenylyl cyclase-associated protein 1 (CAP1)	52222	8,27	88	31%	0,72	0,8	0,48

22	EH domain-containing protein 1 (Testilin)	60646	6,35	118	29%	2,32	1,47	1,36

23	far upstream element-binding protein 2 (FUBP2)	73063	8,02	77	17%	3,62	3,36	3,08

24	far upstream element-binding protein 2 (FUBP2)	73063	8,02	56	9%	0,01	0,01	0,01

25	6-phosphogluconate dehydrogenase (6PGD)	53619	6,8	56	20%	2,06	1,82	2,09

26	alpha-enolase (ENOA)	47481	7,01	61	37%	2,02	2,61	1,02

27	catalase	59947	6,9	102	40%	0,62	0,98	0,48

28	elongation factor 2 (EF-2) isoform 2	96246	6,41	70	14%	1,46	1,38	2,56

The MS spectra of protein digests were used to search the NCBInr database using the ProFound™ search engine. Protein names been assigned according to the Swiss-Prot database. Differentially expressed proteins (ratio > 2) that were up-regulated or down-regulated in response to LPS, mCRP or pCRP for 18 h are listed. The LPS, mCRP and pCRP columns correspond to the expression of each protein relative to its expression in control. Results presented are mean values for three independent analyses performed for each condition. The spot numbers (#) are identical to those given in Figure 2. cov: coverage.

**Table 2:** Selected differentially expressed proteins are tabulated with mean values for three independent analyses and standard deviation of spot density.

spot#	Protein	control			LPS			mCRP			pCRP		
		**mean**	**sd**	**CV**	**mean**	**sd**	**CV**	**mean**	**sd**	**CV**	**mean**	**sd**	**CV**

1	HS90beta	12155	911,6	7,5	17762,2	1101,3	6,2	4658,7	195,7	4,2	12939,9	2368,0	18,3

4	IRF8	2857,2	734,3	25,7	988,2	175,9	17,8	923,3	36,0	3,9	1383,2	225,5	16,3

5	HS105 IFa	562,1	169,8	30,2	1133,5	240,3	21,2	1521,4	429,0	28,2	904,5	104,0	11,5

6	HS105 IFb	404,7	100,4	24,8	785	83,2	10,6	578,2	101,2	17,5	1256,2	374,3	29,8

7	ACTN4	971	153,4	15,8	3162,5	294,1	9,3	2584,7	615,2	23,8	3580,3	211,2	5,9

8	HSP70	1474,4	246,2	16,7	5791	1268,2	21,9	5237,8	361,4	6,9	3240,2	3,2	0,1

9	UBE1	1247,9	68,6	5,5	1398,1	423,6	30,3	2500,3	125,0	5	1728,7	34,6	2,0

17	RIPK2	905,2	18,1	2,0	2129,4	166,1	7,8	2198,9	281,5	12,8	2210,1	276,3	12,5

22	EHD1	347,4	40,6	11,7	807,2	17,0	2,1	510,2	34,2	6,7	472,3	91,2	19,3

26	ENOA	1255	25,1	2,0	2540,5	454,7	17,9	3272,3	618,5	18,9	1281,1	234,4	18,3

overall CV(n = 10 proteins)				14,2			14,5			12,8			13,4

ACTN4 and RIPK2 are increased in the LPS, as well as in the m- and pCRP group. ENOA is increased by LPS and mCRP stimulation of THP-1 monocytes.

Overall, the expression profiles of LPS, mCRP and pCRP-treated cells differ significantly from the control cells, as well as from each other. Other proteins that were identified include several transcription-factors and cell metabolism and signal transduction associated proteins (see Table 1.), further indicating an increased transcription and cell metabolism in the treatment groups.

### LPS, but not CRP, mediates effects via NF-κB and MAPKinase-signaling pathways

**Figure 5:**
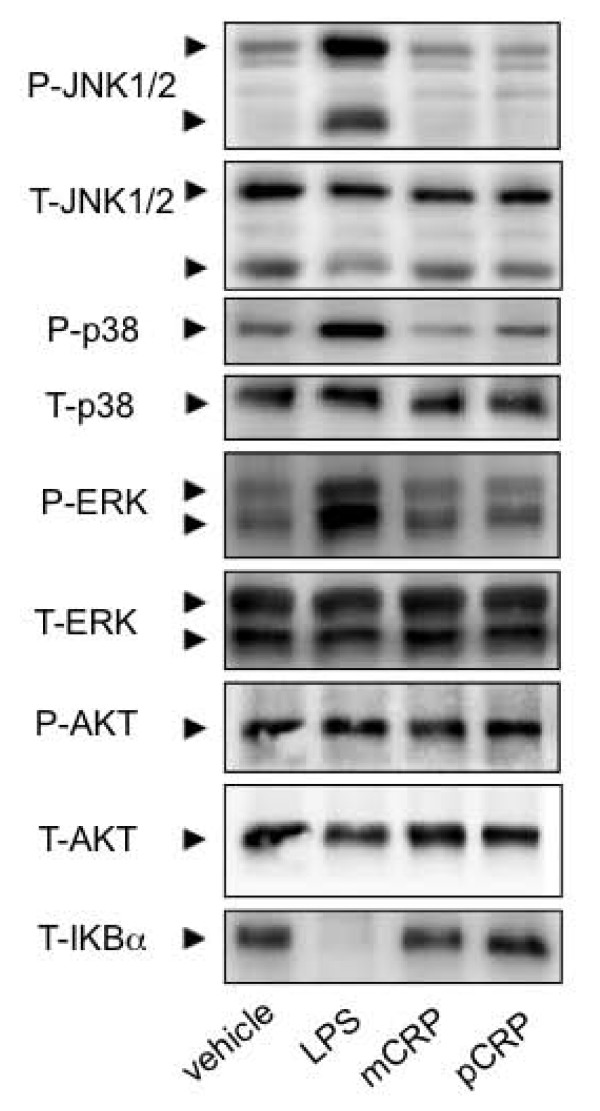
**LPS, but not CRP, mediates effects via NF-κB and MAPKinase-signaling pathways**. In the LPS treated group increased phosphorylation of JNK, p38 and ERK and degradation of IKBα are detected by Western blotting. There is no significant signal transduction in either the mCRP or pCRP treated group through these pathways when compared to control. This result demonstrates that p- and mCRP are not sharing these signal transduction pathways with LPS and that the results of this study are not confounded by LPS contamination.

To investigate the mechanism underlying this difference in expression profile NF-κB and MAPKinase-signaling pathways were investigated. Phosphorylation of key signal transduction enzymes was assessed for each study group. The enzymes studied were c-Jun N-terminal kinase (JNK), p38, extracellular signal-regulated kinase (ERK), AKT8 virus oncogene cellular homolog (AKT) and inhibitor of NF-κB (IKBα). Results are shown in Figure 5.

In the LPS treated group increased phosphorylation of JNK, p38 and ERK and degradation of IKBα are clearly shown consistent with previous studies [8]. However, there was no significant signal transduction in either the mCRP or pCRP treated group through these pathways when compared to control. Whilst this does not demonstrate the signaling mechanism of either CRP group, it is clear that it differs substantially from that induced by LPS. This result is important as it establishes that mCRP dialysis was effective and that these results are not confounded by LPS contamination.

### Validation of proteins by Western blotting

Western blotting using specific antibodies confirmed the protein identity of HSP90, HSP70, RIPK2, UBE1, HSP 105 and aACT4. A selection of the differentially expressed proteins is shown in Figure 5. These results further confirm the identity of the proteins as identified by mass spectrometry.

### Monitoring the spontaneous pCRP dissociation on polystyrene flask

**Figure 6:**
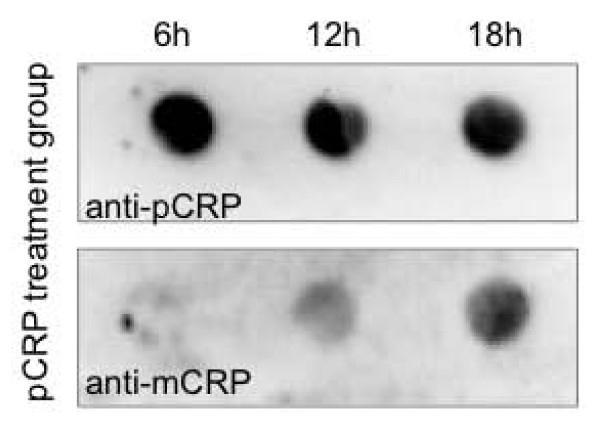
**Incubation of pCRP in polystyrene flasks leads to a partial dissociation of the protein to mCRP**. After various hours of incubation time samples of the incubation assays with pCRP were dot-blotted and the blots probed with mCRP (clone 9C9) or pCRP (clone 8D8) specific antibodies. Whereas the pCRP staining shows a mild decrease the mCRP staining increases gradually in the course of the experiment.

After various hours of incubation time samples of the pCRP incubation assays were dot-blotted and the plots probed with mCRP (clone 9C9) or pCRP (clone 8D8) specific antibodies (Figure 6). pCRP staining shows a slight decrease in intensity in the course of the experiment, whereas mCRP positivity gradually increases. This suggests a partial spontaneous dissociation of pCRP to mCRP the protein that potentially influences the outcome of the experiment.

## Discussion

The hypothesis to be tested in this study was that mCRP induces specific changes in the protein expression profile of THP-1 cells that potentially represent a pro-inflammatory phenotype. An *in vitro *treatment-control experimental design was used to determine CRP isoform-specific effects on proteins isolated and displayed using 2D SDS PAGE and MALDI Tof/Tof analysis. The three major findings of this study are: (1) mCRP and pCRP both induce changes in the protein expression profile of THP-1 monocytes that can be interpreted as pro-inflammatory. (2) mCRP induces changes that are distinct from pCRP, further supporting the concept of a distinct role of mCRP and pCRP in vascular inflammatory diseases, including atherosclerosis. (3) LPS induced proteomic changes and LPS induced signaling pathways differ from mCRP and/or pCRP induced changes.

The causal role of CRP in the pathophysiology of atherosclerosis is currently an area of great controversy. In particular the existence of two isoforms mCRP and pCRP have complicated the analysis and interpretation of experimental findings. In vitro, pCRP has been reported to increase IL-8 production in monocytes,[9] and to increase monocyte adhesion to human aortic and umbilical vein endothelial cells under static and flow conditions[10,11]. However, several studies have also identified possible anti-inflammatory effects of pCRP [12], and anti-atherosclerotic actions for mCRP [13]. In contrast to pCRP, mCRP induces IL-8 secretion in neutrophils [3] and human coronary artery endothelial cells (HCAEC) [2], promotes neutrophil-endothelial cell adhesion [14], delays apoptosis of human neutrophils [15]. Additionally, both CRP conformations interact differently with components of the complement cascade [16].

Recently, we identified a mechanism by which pentameric CRP is dissociated to monomeric CRP on activated platelets, which is then deposited in atherosclerotic plaques [1,17,18]. These findings support the concept of a causal role for CRP in the development of atherosclerosis that might be independent of CRP as a risk factor in atherosclerosis associated cardiovascular events [19]. In addition, we could show that mCRP induces monocyte activation and adhesion to a far greater extent than pCRP. Based on our previous findings we have investigated the intracellular mechanisms involved resulting in this current study.

The result of our proteomic analysis suggests that mCRP has pro-inflammatory properties at the proteomic level, recognizing the limitations of proteomic data in predicting the functional relevance of up-regulation of single proteins. One of our key findings is the up-regulation of the ubiquitin-activating enzyme E1 (UAE1) by mCRP, but not pCRP or LPS. This is of particular interest as the role and the stimulation of the ubiquitin-proteasome system (UPS) in atherosclerosis has been suggested previously [20]. The UPS consists of several enzymes which function to degrade proteins that are presented by helper proteins, so called chaperones. These chaperones assist in the post-translational folding of proteins to their correct 3-D-structure, but also recognize mis-folded proteins and present them for degradation by the UPS [20,21]. Thus, the UPS-chaperone system assures the quality of intracellular proteins. The coronary artery plaques associated with fatal acute myocardial infarction are characterized by increased expression of ubiquitinated proteins [22]. Similarly, carotid artery plaques from patients with symptoms of focal cerebral ischemia display a higher level of ubiquitinated proteins than those obtained from asymptomatic patients [23,24].

The up-regulation of UAE1 in mCRP stimulated monocytes may also be a sign of the induction of the macrophagic differentiation process induced by mCRP, as a crucial role for UAE1 in the differentiation process and the associated nucleotide excision repair has been recently described [25].

Both LPS [26] and mCRP [1] can induce reactive oxygen species (ROS) in monocytes or THP-1 monocytes. Therefore it is unlikely that the proteomic changes observed in the treatment groups are mediated by ROS as there is a substantial difference in the proteomic profile of THP-1 cells treated with LPS and those treated with mCRP. However, the induced oxidative stress can lead to an increase in the expression of chaperones, including those of the family of heat shock proteins (HSPs). One of the HSPs that is most closely associated with atherosclerosis is HSP 70, which is expressed in atherosclerotic lesions [27]. In our proteomic analysis we found a significantly increased expression of HSP70 in monocytes stimulated with mCRP compared to the pCRP treated cells and PBS treated control cells. In experimental models of ischemia and reperfusion injury it has been suggested that HSP 70 mediates myocardial protection [28,29]. Furthermore, HSP70 has been found to be up-regulated in the proteome of monocytes in acute coronary syndrome (ACS) [30], a disease in which circulating CRP levels are increased as well. Our results suggest that there is a potential interaction of HSP 70 with CRP that warrants further research.

Other HSPs differentially expressed are members of the HSP 105 family. HSP 105 alpha and beta are alternatively spliced products derived from the HSP 105 gene transcript. HSP 105 alpha is constitutively expressed but transcription is increased in response to various stressors. In contrast HSP 105 beta, which lacks 44 amino acids from HSP 105 alpha, is specifically expressed during mild heat shock [31]. These proteins can exist as complexes associated with HSP 70 in mammalian cells [32]. Similar to HSP 70, HSP 105 alpha and beta suppress the aggregation of denatured proteins caused by heat shock in vitro. It also has been suggested that HSP 105 alpha and beta can function as a substitute for HSP 70 to suppress the aggregation of denatured proteins in cells under severe stress, in which cellular ATP levels are elevated [33]. Overall, the up-regulation of these proteins could potentially indicate cellular stress, induced by CRP stimulation of THP-1 monocytes. Interestingly, the differential expression of HSP 105 alpha is induced by mCRP, whereas pCRP induces expression of HSP 105 beta, further supporting our concept of discrete function and effects of mCRP and pCRP.

Other proteins increased in the treatment groups that are associated with the inflammatory response include alpha-enolase, alpha-actinin 4 (ACTN4) and EH domain containing protein 1 (EHD1 = Testilin). Alpha-enolase is significantly and uniquely increased after mCRP treatment. Alpha-enolase serves as a plasminogen receptor on macrophages and therefore has an important function in inflammatory cell recruitment [34]. In respect to the development of atherosclerosis, cholesterol loading of macrophages induces expression of alpha-enolase in mice [35].

ACTN4, as a non-muscle alpha-actinin, is functionally important for leukocyte diapedesis in endothelial cells [36] and as such important for cell motility in inflammation. In addition, it has been detected in lipid rafts of THP-1 monocytes, and an involvement in vesicular trafficking and phagosome formation has been suggested [37].

EHD1 regulates the endosomal transport of beta-1 integrins [38], which are important cell adhesion receptors involved in adhesion and transmigration of inflammatory cells [39]. It is significantly up-regulated in LPS stimulated THP-1 monocytes, but not in cells incubated with CRP isoforms further underlining the differentiated proteomic response of these cells to LPS versus CRP co-incubation that cannot be attributed to the contamination of the CRP preparations with LPS, as suggested for other experiments [40].

Overall, the proteins up-regulated by the CRP isoforms potentially represent the induction of a pro-inflammatory monocytic phenotype for adhesion cell recruitment and transmigration into the area of inflammation.

A recent comprehensive genomic study of human primary monocytes by Hanriot et al. [12] demonstrated complex cellular responses, both pro- and anti-inflammatory, following stimulation by (p)CRP. Amongst other findings chemokines (Gro protein alpha and beta, Interleukin (IL)-8), cytokines (IL-1 alpha and beta, IL-6, IL-19), integrins (Integrin alpha 6 and beta 1), Liver X receptor alpha and MAPK 8 were all up-regulated. Interestingly these authors also found a down regulation of several pro-inflammatory chemokines, suggesting potential anti-inflammatory effects of (p)CRP in primary monocytes at the genomic level.

Our proteomic dataset derived from a monocytic cell line complements these findings. The increased expression of Alpha-enolase and ACTN4 identified in our work is consistent with the up-regulation of several genes discussed above that encode for proteins involved in leukocyte trafficking, adhesion and transmigration by Hanriot et al. [12]. The increased expression of these genes and proteins potentially characterizes a pro-inflammatory phenotype induced by (p)CRP.

CRP is generally believed to bind to receptors of the Fc gamma Receptor family [41]. Recently it has been shown that lipid rafts are involved in mCRP signal transduction [1,42]. The proteomic expression model used in this study involving prolonged incubation periods was not able to identify a discrete receptor ligand interaction. Successful resolution of this question will most likely require development of dedicated cell models and remains an area of active ongoing research.

One potential limitation of our study is the use of THP-1 cells, instead of primary monocytes. THP-1 cells could potentially reveal different properties compared to the primary cells. However, THP-1 cells have been previously used as a surrogate for primary monocytes, particularly in proteome research, and their use has so far contributed towards several pathophysiologically important findings, specifically in the field of atherosclerosis research [43]. Therefore, although our findings must be interpreted with this in mind it does not invalidate this data.

Compared to our previous findings, in which pCRP showed significant less pro-inflammatory properties compared to mCRP, our findings in this proteomic study are surprising, as pCRP induces some pro-inflammatory proteomic changes as well. This may be due to one of the following factors. In our previous work we examined the short-term effects of the two isoforms on monocytes, which is fundamentally different from the 18 hours incubation period that is necessary to evaluate proteomic changes in the set of experiments described herein. Secondly, as the incubation was carried out in polystyrene tissue culture dishes spontaneous dissociation of pCRP to mCRP may occur over the course of the experiment. Indeed such dissociation has been described previously on polystyrene materials [44] and was confirmed by us. Therefore some effects seen in pCRP treated cells may indeed be effects caused by mCRP, which could potentially influence the outcome of the experiments and warrants further investigations into the distinct properties of the two isoforms.

## Conclusions

This study confirms the distinct properties of mCRP and pCRP. In particular, mCRP related changes of the THP-1 proteome are mainly associated with the heat-shock response. These results are consistent with a pathophysiological role of mCRP in inflammatory disease and identify a number of novel target proteins that are involved in the changes occurring in monocytes upon activation by CRP isoforms. These potentially represent novel therapeutic targets and may be of particular relevance for monocyte activation by CRP in the vicinity of atherosclerotic plaques and in the potential of therapeutic targeting of mCRP in vascular disease.

## Methods

### Culture, stimulation and harvesting of THP-1 cells

Human THP-1 cells were grown in RPMI 1640 medium containing 10% fetal bovine serum (FBS), 2 mM glutamine, 10 mM HEPES, 1 mM sodium pyruvate, 561025 M b-mercaptoethanol, penicillin (100 U/mL), and streptomycin (100 mg/mL) and stored at 37°C in an atmosphere of 5% CO_2_.

Cells were seeded at a density of 6 × 10^5 ^cells/mL in medium containing 10% FBS. The cells were pre-incubated in serum-free medium for 24 h and then treated with PBS, mCRP (25 μg/mL), pCRP (25 μg/mL) or 100 ng/ml LPS (Sigma) for 18 h. mCRP was prepared as described previously [1], pCRP was from Calbiochem and dialyzed extensively against PBS prior to use.

### Intracellular signaling analysis

Human THP-1 cells were grown as described above. Cells were seeded at a density of 1×10^6 ^cells/mL in medium containing 3% FBS for 24 hours prior to treatment with PBS, mCRP, pCRP or LPS as above. Initial experiments were performed at multiple time points (0, 15, 30, 60 or 120 minutes) to assess when activity changes could most reliably be found. For all published data experiments were ceased at 60 minutes. After stimulation cells were collected, pelleted by centrifugation at 3,000 G for 5 minutes, washed in 4°C phosphate buffered saline (PBS) and pelleted again. PBS was then removed and cells were lysed for 10 minutes at 4°C in 80 μL lysis buffer containing 50 mM Tris, 130 mM NaCl, 5 mM EDTA, 1% NP-40, 1 mM PMSF, 1 mM NaF, protease and phosphatase inhibitor. Cellular debris was pelleted by centrifugation at 16,000 G for 30 minutes at 4°C. Supernatant was then collected, protein concentration measured by BCA at 570 nm and stored at -80°C prior to further analysis.

### 1D SDS Polyacrylamide Gel Electrophoresis (SDS PAGE)

30 ug of measured protein sample was combined with Laemmli buffer, denatured for 5 minutes at 95°C and loaded on to 10% SDS PAGE gels. Samples underwent semi-dry transfer to nitrocellulose membrane and were probed for alteration in gene expression as shown. Phosphorylated and total protein loads are shown for each kinase except IKBα, which is ubiquitinated and degraded following activation. All antibodies were obtained from Cell Signaling Technology, USA. Results shown are representative blots performed in triplicate of individual experiments which were each performed at least three times.

### Sample Preparation

Cells were harvested at 1400 g for 10 min and washed 3 times with PBS. Cell pellets were lysed using 4% CHAPS, 7 M urea, 2 M thiourea, supplemented with 4.6 mM PMSF, 25 μg/mL RNAse (MP Biomedicals, NSW, Australia), 100 μg/mL DNAse (MP Biomedicals), 44 mM MgCl_2_. Total protein content of each sample was determined using the Coomassie^® ^Plus Protein Assay Reagent Kit (Pierce Biotechnology, USA).

### Two-Dimensional SDS Polyacrylamide Gel Electrophoresis (SDS PAGE)

A total of 50 μg protein was loaded onto first dimension Ready Strip™ gels (11 cm, pH 5-8; Bio-Rad Laboratories, Hercules, CA, USA) following sample solubilisation with 200 μL of multiple chaotrope solubilisation solution (7 mol/L urea, 2 mol/L thiourea, 100 mmol/L dithiothreitol, 4% CHAPS, 40 mmol/L Tris buffer, 0.5% carrier ampholytes pH 3-10, trace bromophenol blue). Strips were actively rehydrated at 50 V at 20°C for 16 h. Proteins were isoelectrically focused at 250 V for 15 min and then slowly ramped to 8000 V for 150 min and then held at 8000 V for a total of 35 000 Vh/gel (i.e., a total of 42 000 Vh per gel). The IPG strip was equilibrated twice for 15 min with gentle agitation in 6 mL of equilibration solution (0.1% Tris-HCl (pH 6.8), 5.5 M urea, 0.3% glycerol, 0.035 M SDS and 0.065 M DTT). DTT was replaced with iodoacetamide (14 mM) and traces of Bromophenol Blue for the second equilibration solution. Second dimension separation was performed on precast 10.5-14% Tris-HCl Criterion Gels (Bio-Rad Laboratories). Gels were electrophoresed at 10 mA/gel for 1 h, 20 mA/gel for 2 h and then 30 mA/gel for 30 min. Gels were then fixed in methanol/acetic acid (10%) for 1 h then stained in SYPRO Ruby^® ^fluorescent protein dye (Bio-Rad Laboratories) at room temperature for 16 h on a rocking platform. Gels were destained for 1 h in methanol/acetic acid (10%/7%). Gels were individually scanned using an FX imager with a 532-nm laser at 100 μm resolution (Bio-Rad Laboratories). The protein spots were detected, quantified, and matched using PDQuest V7.3.1 (Bio-Rad Laboratories). Each gel was normalized to the total quantity of valid spots. The gels were then counterstained with colloidal Coomassie brilliant blue (CBB) to enable visualization and manual excision. 2D-PAGE and cell seeding was performed in triplicate for each treatment.

### In-Gel Digestion and Peptide Analysis Using Matrix-Assisted Laser Desorption-Ionization Time-of-Flight Mass Spectrometry

In this study, a MALDI Autoflex II, Bruker Biosciences mass spectrometer was used to estimate peptide masses generated from the in-gel digestion of the 2D PAGE protein spots. The database search results and tabulated protein identifications are from data collected from the mass spectrometer. Only high scoring and confident hits retrieved from the selected databases using Mascot search engine are tabulated. Selected protein spots were manually excised from colloidal CBB-stained 2D gels using a spot picker (The Gel Company, San Francisco, CA, USA, picker head, 1.5-mm diameter). The gel plugs were rinsed by transfer into 96-well plates (Greiner Bio-One BioScience, NC, USA) containing deionized water (100 μl per well, MilliQ grade). Digestion using trypsin with subsequent application to a Matrix Assisted Laser Desorption-Ionization Time-of-Flight (MALDI-ToF) target plate (Bruker Daltronics, Bremen, Germany) was performed robotically with an Ettan Spot Handling workstation (GE Healthcare, Uppsala, Sweden). The gel plugs were destained in 100 μl of 50 mM ammonium bicarbonate (Riedel-de Haen, Germany)/50% methanol (Lab-Scan Analytical Sciences, Gliwice, Poland) for 30 min, then washed three times in deionized water (100 μl, 5 mins, Milli Q grade). Gel plugs were then incubated in 100 μl of 50 mM ammonium bicarbonate/50% methanol for 30 min followed by incubation in 100 μl of 75% acetonitrile (ACN, Lab-Scan Analytical Sciences, Ireland) for a further 30 min. After drying the gel plugs (40°C, 23 min), 10 μl of sequencing grade trypsin solution (20 ng/μl, Promega, Madison, WI, USA) dissolved in 20 mM ammonium bicarbonate was added and incubated (37°C for 60 min). During the following extraction step, gel plugs were covered with 60 μl 0.1% trifluoroacetic acid (TFA, Pierce, Rockford, USA)/50% ACN and incubated for 20 min. The peptide containing supernatant was transferred to a new 96-well plate, and the extraction was repeated (40 μl). The supernatants were pooled and dried (40°C for 3 h). The peptides were resolubilized by manually adding 50% ACN/0.1% TFA (15 μl) into each well and gently vortexing (10 mins, Vortex Genie 2). The resolubilized peptide mixtures (0.3 μl) were each combined with MALDI matrix (1.0 μl, α-cyano-4-hydroxy cinnamic acid, 3 mg/ml in acetone/ethanol (1:2, v/v), Bruker Daltronics) and applied (1.3 μl) to the Anchorchip target plate using the Ettan Spot Handling robotic workstation (GE Healthcare). The peptide-matrix samples were allowed to dry on the target plates (10-15 min) before data collection.

### MS Data Collection, Database Searching, and Protein Identification

General instrument settings for the Autoflex II MALDI-ToF/ToF (Bruker Daltronics) are as follows: mode, positive, and reflector; pulsed ion extraction, 120 ns; laser intensity, 22-28%; laser frequency, 25 Hz; 600 laser shots were collected and summed for all MS data. The mass range (800-3,000 m/z) of the mass spectrometer was internally calibrated using the autolytic peaks of trypsin (842.510 and 2,211.104 m/z). Matrix was suppressed using a high-gating factor. Signal suppression below 800 m/z was activated. Data acquisition and processing was performed using instrument-specific software, Flex-Control, FlexAnalysis, and BioTools software (Bruker Daltonics). Peaklists were generated before database searching. Data collected from the Autoflex II MALDI ToF/ToF, was submitted to the SwissProt database (release 51.0, 31October 2006) using the Mascot search algorithm (Matrix Science, http://www.matrixscience.com). Typical search parameters were as follows: mass tolerance, 0.5 Da; missed cleavages, 2; enzyme, trypsin; fixed modifications, carbamidomethylation; variable modification, Oxidation (M); taxonomy, Homo Sapiens.

### Validation of proteins by Western blotting

#### Antibodies and reagents

Monoclonal antibodies against Hsp90 were obtained from BD Biosciences (California, USA), RIPK2 (clone 7F5) Abnova (Taiwan) Corporation and αACT4(7H6) Abcam (Cambridge, UK). Polyclonal antibodies against HSP105 was obtained from MBL (Woburn, MA, USA), UBE1 from Atlas Antibodies (Stockholm, Sweden) and Hsp70(Hsp72) from Stressgen (MI, USA). Horseradish peroxidase-conjugated goat anti-mouse antibody was obtained from Bio-Rad Laboratories while HRP-conjugated donkey anti-rabbit antibody was from Amersham Biotechnology (Uppsala, Sweden).

The identity of all proteins of interest from mass spectrometry data were confirmed by Western blotting and probing with specific antibodies. All samples were reduced with 2% beta-Mercaptoethanol and 20 μg/sample was applied to 10% criterion pre-cast gels (BioRad Laboratories). Resolved proteins were transferred to nitrocellulose membranes and probed with primary antibody followed by peroxidase-labelled secondary antibody and visualized by enhanced chemiluminescence (ECL) (Amersham, Buckinghamshire, UK) detection system according to the manufacturer's instructions. Dual precision MW marker (BioRad Laboratories) was used to determine the correct band size. Band density was evaluated by Quantity One software (BioRad Laboratories).

### Monitoring CRP dissociation by dot-blotting

Samples of the incubation experiments were taken after the 6, 12 and 18 hours of the experiment and dot-blotted by a BioRad 96-well Dot Blot apparatus (Bio-Rad Laboratories) onto nitrocellulose membranes according to the manufacturers instructions. Membranes were probed for mCRP expression by mCRP specific antibodies (clone 9C9) as described previously [1].

## Competing interests

The authors declare that they have no competing interests.

## Authors' contributions

SUE conceived of the study, designed and carried out experiments, analyzed data and wrote the manuscript. JH designed experiments, performed experiments and wrote the manuscript, KO carried out the 2D-gels and wrote the manuscript. MA performed experiments. GIL designed and performed experiments. KJW conceived of the study, and participated in its design. HB designed and analyzed experiments. GE R participated in the design and coordination of this study and wrote the manuscript, KP conceived of, designed and coordinated the study, and wrote the manuscript. All authors read and approved the final manuscript.
